# On the Treatment of Tetanus by Liquor Potassæ

**Published:** 1861-04

**Authors:** Joseph Reid


### *On the Treatment of Tetanus by Liquor Potassæ.* By JOSEPH REID, M. D.

Having lately had under my care a case of tetanus, in which the treatment I adopted proved successful, and feeling that another opportunity may not offer in my practice to test more fully the effects of the same remedy in the same disease, I am anxious that other practitioners may be induced, from a short statement of the case, to try that which I found in this instance to be so highly efficacious. I regret much that I did not take daily notes of the case; but the following brief report will, I trust, be sufficient evidence of the practice and its results.

Mr. S——, aged thirty, of active habits, delicate constitution, and possessing a nervous temperament, came under my care June first, 1860. States that a few days since he was exposed to a cold easterly wind for some hours when driving in an open vehicle; but that he did not observe any ill effects therefrom, and felt in his usual health until this morning, when he for the first time, experienced a sense of constriction and tightness in the throat, but which symptoms appeared to diminish until mid-day, when they became suddenly aggravated, causing him serious alarm, and obliging him to desist from business and seek medical relief. When he visited me, I found him in a state of extreme excitement, in momentary dread of strangulation; he can not swallow the smallest portion of solid food, and when he endeavored to drink, the act of deglutition was attended with such a sense of suffocation and anxiety, that it was with much difficulty I could induce him to make the attempt. At this period there was no other symptom present so that it was impossible to determine the exact nature of the attack; but by degrees the disease became developed; the countenance assumed the tetanic expression; the masseter muscles and those of the cervical region were now firmly contracted, the sterno-cleido mastoid standing out like tense cords; the lower jaw became almost fixed; tongue could with difficulty be protruded between the incisor teeth, and at times was spasmodically darted forwards, so as to run a danger of severe laceration. The walls of the abdomen likewise became extremely hard and rigid, and much difficulty and distress was experienced in passing urine, which could only be voided at long intervals, although a desire to do so was seldom absent. Pain was also complained of, shooting from the ensiform cartilage through the diaphragm towards the vertebral column, and the spasmodic jerks from which the patient suffered were so intense and frequent, as to deprive him of rest both night and day. His bowels were obstinately constipated, and irritability of the stomach, with vomiting, added much to his general distress. His skin was bathed in profuse perspiration. The pulse, which at times was intermittent, was also irregular as to its frequency, an extreme variation often occurring within a few hours.

Such were the symptoms present, and such was the condition of the patient on the eighth day from the commencement of the attack; and as the case appeared to be rapidly growing from bad to worse, I thought it necessary to alter the treatment from that which I had been pursuing—namely, mercury, with anodynes and antispasmodics—to what I considered might be attended with more success. I accordingly prescribed liquor potassæ which I ordered in combination with syrup of poppies and camphor-water, after a few doses of which I was much pleased to find a marked improvement in the most urgent of symptoms, the earliest amendment being a perfect freedom from the difficulty of urine. The stomach soon became settled and capable of retaining nourishment. By degrees the tension of the muscles, and the spasmodic jerkings became diminished. The bowels acted without the assistance of enemata, which were before absolutely necessary, and in every particular, progress towards health followed the employment of this medicine.

Such are the outlines of this case; and I consider it may be satisfactory if I state my reasons for selecting the liquor potassæ. But first I may refer to the very uncertain and scanty information which we derive from pathological appearances, even when such appearances are present; for often the most careful examination fails to discover any alteration of structure which can be looked upon as the effects of tetanus only. No doubt there is sometimes found a vascularity of the mucous membrane of the œsophagus, and of that covering the cardiac orifice of the stomach; at others, there is discovered some effusion within the cranium or spinal canal; but all or any of these are not always present, and are, perhaps, more frequently so after death from other causes. The disease cannot be one of an inflammatory character for whether it terminates fatally after a few days or some weeks, there is not, as a rule, any change left which can be considered as a product of inflammation or which, if looked upon as such, bears the least ratio to the severity of the symptoms during life. But this very absence of morbid appearances leads me to suppose that mere functional disturbance in the operation of the nervous and muscular systems may constitute a principal, if not the entire, of the disease. When we remember how intimately incorporated are the capillary vessels and their contents with the medulla of the nerve and the fibrillæ of the muscle, it is easy to conceive how muscular contraction may follow on any irritation of the nerve-fibre by causing an accumulation of fluid in the vessels of the contracting muscle; and although it may be impossible to define the exact proportion in which the blood, the nerve, and the tonicity of the muscle may be engaged in tetanus, yet it appears to me that the rigidity of the last is different from the contraction which we are accustomed to find in other spasmodic disease: it seems to resemble more the rigor mortis, or, death-stiffening—a condition in which the influence of innervation is absent. But superaded to this, we observe in tetanus the alternating movements of contraction and relaxation, arising no doubt, from reflex action operating through the nervous centres. To obtain a less easily excited condition of this system, also a diminution in the tonicity of the muscular fibre, and a decrease in that portion of the blood through whose agency is supplied irritability or vital activity to the nervous and muscular structures, would be to gain a certain control over an exciting cause, and a relaxation in the leading features of the disease; and believing that in liquor potassæ we have a remedy calculated to effect these changes, I prescribed it in the foregoing case, at a time when all the symptoms were so urgent as to afford but slight hopes of recovery and it is owing to the very rapid improvement which followed on its administration that I feel a further trial of liquor possæ in tetanus may be attended with like success.—*London Lancet.*

